# Targeting Tuberculosis and HIV Infection-Specific Regulatory T Cells with MEK/ERK Signaling Pathway Inhibitors

**DOI:** 10.1371/journal.pone.0141903

**Published:** 2015-11-06

**Authors:** Nora V. Lieske, Kristian Tonby, Dag Kvale, Anne M. Dyrhol-Riise, Kjetil Tasken

**Affiliations:** 1 Centre for Molecular Medicine Norway, Nordic EMBL Partnership, University of Oslo, Oslo, Norway; 2 Institute of Clinical Medicine, University of Oslo, Oslo, Norway; 3 Department of Infectious Diseases, Oslo University Hospital, Oslo, Norway; 4 Kristian Gerhard Jebsen Inflammation Research Centre, University of Oslo, Oslo, Norway; 5 Biotechnology Centre, University of Oslo, Oslo, Norway; University of Hawaii, UNITED STATES

## Abstract

Human regulatory T cells (Tregs) are essential in maintaining immunological tolerance and suppress effector T cells. Tregs are commonly up-regulated in chronic infectious diseases such as tuberculosis (TB) and human immunodeficiency virus (HIV) infection and thereby hamper disease-specific immune responses and eradication of pathogens. The MEK/ERK signaling pathway is involved in regulation of the FoxP3 transcription factor, which directs a lineage-specific transcriptional program to define Tregs and control their suppressive function. Here, we aimed to target activation of disease-specific Tregs by inhibition of the MEK/ERK signaling pathway based on the hypothesis that this would improve anti-HIV and anti-TB immunity. Stimulation of T cells from untreated TB (n = 12) and HIV (n = 8) patients with disease-specific antigens *in vitro* in the presence of the MEK inhibitor (MEKI) trametinib (GSK1120212) resulted in significant down-regulation of both FoxP3 levels (MFI) and fractions of resting (CD45RA^+^FoxP3^+^) and activated (CD45RA^−^FoxP3^++^) Tregs. MEKI also reduced the levels of specific T effector cells expressing the pro-inflammatory cytokines (IFN-γ, TNF-α and IL-2) in both HIV and TB patients. In conclusion, MEKIs modulate disease antigen-specific Treg activation and may have potential application in new treatment strategies in chronic infectious diseases where reduction of Treg activity would be favorable. Whether MEKIs can be used in current HIV or TB therapy regimens needs to be further investigated.

## Introduction

Regulatory T cells (Tregs) are key players in maintaining immune homeostasis that ensure immunological self-tolerance as well as protection from auto-immunity and chronic inflammatory diseases [1–4]. Their suppressive function can be exerted via a set of contact-dependent and contact-independent mechanisms and generally results in the down-regulation of effector T cell activation and proliferation [5]. The major regulator of Treg suppressive function is the forkhead box P3 (FoxP3) transcription factor [6, 7] which initiates a lineage-specific gene expression program by acting either as a transcriptional activator or repressor in Tregs [8, 9]. Furthermore, based on earlier observations from our laboratory, Treg activation and up-regulation of FoxP3 expression upon antigen-stimulation depends on the MEK/ERK signaling pathway [10].

Persistent immune activation is a hallmark of chronic infectious diseases such as tuberculosis (TB) and human immunodeficiency virus (HIV) [11, 12]. Tregs protect tissue from damage caused by infection induced inflammation, but at the same time suppress effector T cell immune responses and facilitate pathogen persistence [13].

In TB infection, Tregs proliferate and accumulate at sites of active inflammation and Treg numbers are increased in blood of patients with active TB [14–16]. Furthermore, FoxP3 gene expression is reported to be 2.8-fold higher in Tregs from TB patients compared to healthy individuals [17]. In HIV infection, chronic immune activation and inflammation lead to exhaustion of the immune regenerative capacity and a decline in CD4^+^ cells [11]. Tregs play an important role in chronic viral infections by limiting the immune activation and pathogen-specific immune responses [18]. Although the total number of CD4^+^ T cells and Tregs are decreased during HIV infection, there is a relative increase of Tregs during progression of HIV disease [19]. Thus, depending on the phase of infection, Tregs may play different roles in HIV pathogenesis; while they control viral replication in early infection, they potentially have a negative impact on immune responses in later stages [20].

Globally, 9 million cases of TB disease and 1.5 million deaths from TB were reported in 2013 [21]. One fifth of the previously treated TB cases with recurrent TB disease have multi-drug resistant (MDR)-TB, and extensively drug-resistant TB (XDR-TB) has been reported by 100 countries [21]. MDR-TB and XDR-TB have very restricted treatment options and research into new treatment modalities is needed. Likewise, antiretroviral therapy (ART) does not normalize CD4^+^ counts, T cell activation and dysregulation in many HIV patients compared to uninfected individuals [22]. Thus, targeting immune homeostasis may be a therapeutic strategy for patients with incomplete normalization of CD4^+^ counts (immunological non-responders).

Several clinical trials have investigated the use of MEK inhibitors (MEKIs) for single-agent or combination therapies in different cancer diseases (reviewed in [23, 24]). In contrast, there are few reports on the effects of MEKIs in infectious and inflammatory diseases in general [25]. In this study we tested the hypothesis that inhibition of MEK-dependent up-regulation of FoxP3 and suppressive function in Tregs could improve disease-specific immune responses in the two different chronic infectious diseases, TB and HIV. We found a significant down-regulation of FoxP3 levels in resting Tregs (rTreg) and activated Tregs (aTregs) in response to *in vitro* treatment with MEKI in cell cultures, both from patients with TB and patients with HIV. The effects on effector T cell responses were more differential, but a general decline in the pro-inflammatory cytokines TNF-α, IL-2 and IFN-γ was seen.

## Materials and Methods

### Study participants and processing of samples

Patients with newly diagnosed, untreated active TB disease (n = 12) and ART-naïve HIV positive individuals (n = 8) were recruited from the Department of Infectious Diseases, Oslo University Hospital, Oslo, Norway. Table 1 summarizes demographic and clinical characteristics of both groups. Peripheral blood mononuclear cells (PBMC) were isolated in cell preparation tubes (CPT Becton Dickinson, BD) with sodium heparin, and analysed immediately or cryopreserved in 90% fetal calf serum (FCS, Sigma)/10% dimethyl sulfoxide (DMSO) and stored at -145°C until analysis. Written informed consent was obtained from all participants. The study was approved by the Regional Committees for Ethics in Medical Research (REK-Sør-Øst).

**Table 1:** Patient characteristics.

	TB (n = 12) Median [IQR]	HIV (n = 8) Median [IQR]
Age in years	29 [22–61]	45 [29–54]
Female [% of total]	5 [42]	1 [13]
Origin [% of total]		
Africa	6 [50]	2 [25]
Asia	5 [42]	0 [0]
Europe	1 [8]	6 [75]
Localisation [% of total]		
Pulmonary	6 [50]	-
Extrapulmonary^a^	2 [17]	-
Pulmonary + extrapulmonary^a^	4 [33]	-
IGRA positive^b^ [% of total]	12 [100]	-
CD4^+^ T cell count [x 10^6^/l]	-	557 [333–965]
CD8^+^ T cell count [x 10^6^/l]	-	1151 [830–1915]
HIV RNA in plasma [copies/ml]	-	8900 [910–110000]
ESR^c^	23 [7–88]	4 [1–14]

^a)^ Lymphnode, pericardial, abdominal, cutaneous abscess, osteomyelitis.

^b)^ QuantiFERON-TB Gold^®^.

^c)^ Erythrocyte Sedimentation Rate.

### Reagents

Directly conjugated monoclonal antibodies for staining T cell surface markers were directed to CD3-PerCP (cat. no. 345766), CD4-BV605 (cat. no. 562658), CD25-BV421 (cat. no. 562442), CD45RA-APC-H7 (cat. no. 560674) and CD45RA-V450 (cat. no 560362); antibodies for intracellular staining were FoxP3-Ax488 (cat. no. 560047), IL-2-PE (cat. no. 559334), IFN-γ-PECy7 (cat. no. 557844) and TNF-α-APC (cat. no. 340534); antibodies for sorting of rTregs were CD4-PerCP (cat. no 550631), CD25-PECy7 (cat. no 557741) and CD45RA-PE (cat. no 555489), cell viability was analyzed with 7AAD (cat. no 559925), all from Beckton Dickinson (BD, Biosciences, San Jose, CA, USA). To inhibit protein transport from the Golgi apparatus, Brefeldin A (cat. no B7651) from Sigma Aldrich (St Louis, MO, USA) was used.

MEK inhibitors used were FR180204 (cat. no 203945) from Santa Cruz (Dallas, TX, USA), PD098059 (cat. no. P215) from Sigma-Aldrich, U0126 (cat. no. CALB662005-1) from Calbiochem (Darmstad, DE), CI-1040 (cat. no. MEK-CI-5), AZD6244 (cat. no. MEK-SELU-5) from JS ResearchChTrading (Wedel, DE), PD0325901 (cat. no. 1408) from Axon (Groningen, NL), and MEK162 (cat. no. CT-A162) and GSK1120212 (CT-GSK112) from Chemietek (Indianapolis, IN, USA). Anti-CD3/CD28/CD2-coated MACSiBeads (130-091-441) from Miltenyi (Lund, SE) were used for stimulation of PBMCs from healthy blood donors. Early secretory antigenic target 6 (ESAT-6; 15-mer overlapping peptide pools with >80% purity; Schäfer, Hadsund, DK) and antigen 85 (Ag85) complex (15-mer overlapping peptide with >85% purity; Genscript, Hong Kong, CHN) were used for stimulation of PBMCs from TB patients, whereas complete 15-mer *Gag* and *Env* overlapping peptide panels (NIH AIDS Research and Reference Reagent Program, MD, USA) were used for antigen-specific activation of HIV patient PBMCs. SEB (Staphylococcal enterotoxin B) 0,5 ug/ml (Sigma-Aldrich MO, USA) was used as positive control (data not shown).

### T cell purification and sorting of resting regulatory T cells

Buffy coats from healthy blood donors (n = 3) were obtained from the Department of Immunology and Transfusion Medicine, Oslo University Hospital, Oslo, Norway. CD4^+^ T cells were purified by negative selection using RosetteSep Human CD4^+^ Enrichment cocktail (StemCell Technologies, Grenoble, FR), in combination with Lymphoprep (Medinor, Oslo, NO) according to the manufacturer`s instructions. CD4^+^ enriched T cells were stained with anti CD4-PerCP, anti CD25-PECy7 and anti CD45RA-PE for 30 min on ice. Afterwards, cells were washed once with PBS (2% FCS) and added on a 30 μm filter to ensure a single cell suspension. Cell sorting of rTregs (CD4^+^CD25^+^CD45RA^+^) was performed on a BD FACS Aria II cytometer (488 nm and 633 nm lasers).

### In vitro stimulation of resting Tregs

Sorted resting Tregs were re-suspended at 1 x 10^6^ cells/ml in RPMI medium (RPMI1640 supplemented with 10% FCS, 100 U/ml penicillin, 0.1 mg/ml streptomycin, 1 mM sodium pyruvate and nonessential amino acids) and incubated for 20 min with or without different MEKIs at concentrations ranging from 0.3 nM to 10 μM, prior to stimulation with anti-CD3/CD28/CD2-coated MACSiBeads (bead to cell ratio 1:5). The cells were incubated for 36 hours based on previous studies showing that the peak of Foxp3 up-regulation occurs after 36 h stimulation [10]. Pilot studies of different concentrations of the MEKI were performed to evaluate potential toxic effects, and concentrations up to 10 μM of the MEK inhibitor GSK1120212 (trametinib) proved not to induce cell toxicity during the incubation time (data not shown).

### In vitro stimulation of PBMCs from TB and HIV patients

Frozen PBMCs from HIV and TB patients were thawed, re-suspended in RPMI medium and rested at 37°C overnight. Viability of frozen cells was typically 85%. The next day, cells were incubated with MEKI GSK1120212 at 100 nM (~IC80) and 10 μM concentration for 30 min prior to stimulation with peptide pools of ESAT-6 (2 μg/ml) and Ag85 (2 μg/ml) for TB patients and single peptides *Gag* (2 μg/ml) and *Env* (2 μg/ml) for HIV patients. Cells were then incubated for 36 h and Brefeldin (BFA) (10 μg/ml) was added for the last 10 h to avoid prolonged incubation with potential toxic effects of BFA. SEB (1 μg/ml) was used as positive control for T cell stimulation (data not shown).

### Flow cytometric analysis and data processing

After harvesting, cells were washed in PBS (2% FCS) and then fixed/permeabilized with the FoxP3 staining kit (BD Biosciences) according to the manufacturer`s instructions. Subsequently, cells were stained for intracellular TNF-α, IFN-γ, IL-2 and FoxP3, as well as T cell surface markers CD3, CD4, CD25 and CD45RA, and analyzed on a BD LSA Fortessa cell analyzer (488 nm, 640 nm and 405 nm lasers). For data analysis, FlowJo software (version 10, TreeStar Inc., Ashland, OR, USA) was used. A gating strategy was applied with exclusion of dead cells, debris and doublets. The effects of MEK inhibition were determined by gating on the target population and comparing the fraction of positive cells expressing the various cytokines, the FoxP3 median fluorescence intensity (MFI) and the fraction of FoxP3 positive cells in the respective samples. Resting Tregs (rTregs) were defined as CD3^+^CD4^+^CD45RA^+^Foxp3^+^ and activated Tregs (aTregs) as CD3^+^CD4^+^CD45RA^−^Foxp3^++^ according to Miyara *et al*. [26]. Single, double and triple cytokine producing T cells were delineated by Boolean gating, showing levels of antigen-stimulated PBMCs with or without addition of MEKI.

### Graphical presentation and statistical analysis

Graphical presentations were made using GraphPad Prism (version 6, GraphPad Software Inc., La Jolla, CA, USA) and statistical analyses were performed by using the Wilcoxon matched-pairs signed rank test (two tailed, 95% confidence intervals). Data are expressed as percentiles and interquartile range (IQR). Levels of significance are expressed as p-values (* p< 0.05, ** p<0.01, *** p< 0.001).

## Results

### MEKI interferes with FoxP3 up-regulation upon activation of regulatory T cells

**Fig 1:**
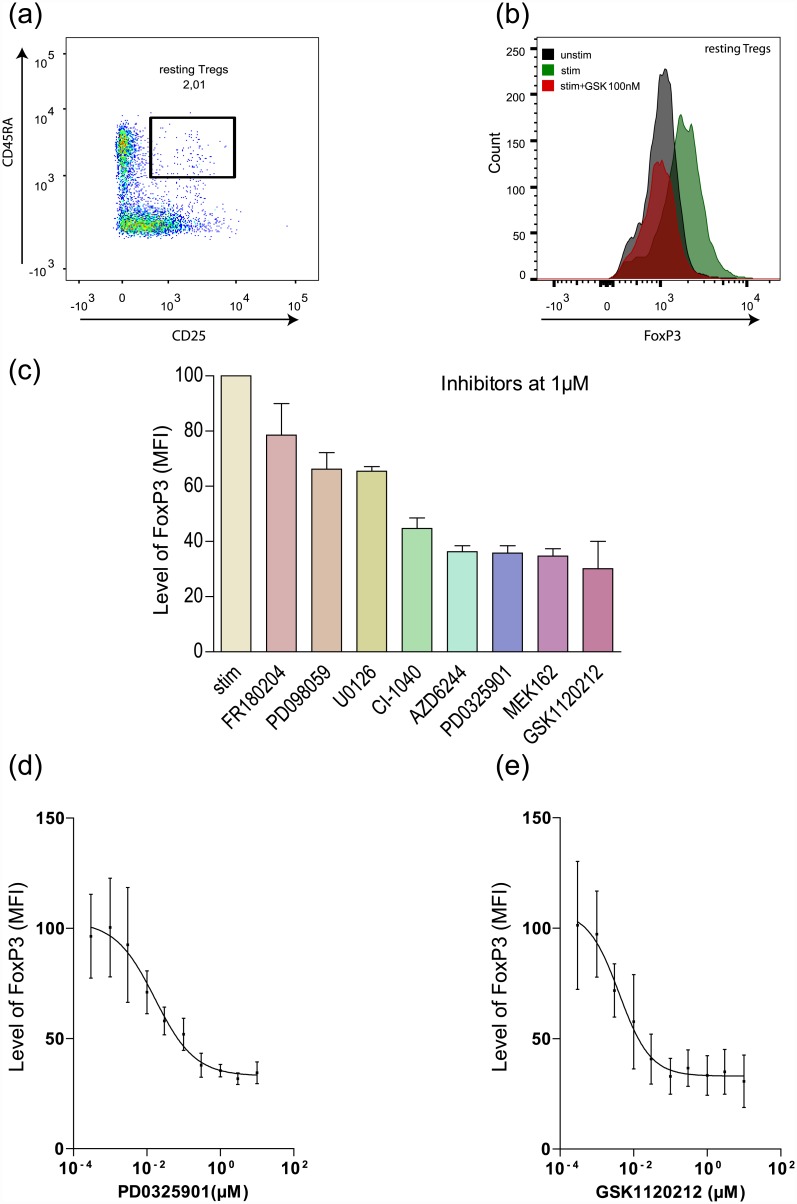
Effect of MEK inhibition on FoxP3 expression in sorted resting Tregs (rTregs). Human blood donor rTregs were stimulated with anti-CD3/CD28/CD2-coated MACSiBeads for 36 h in the presence or absence of MEK specific inhibitors (MEKI), followed by FoxP3 staining and FACS analysis. (a) Gating strategy for sorting of rTregs from CD4^+^ enriched cells. (b) rTregs were stimulated in the presence or absence of the MEK specific inhibitor GSK1120212 or left unstimulated. (c) rTregs were stimulated in presence or absence of the different MEK specific inhibitors FR180204, PD098059, U0126, CI-1040, AZD6244, PD0325901, MEK162, GSK1120212 at 1μM concentration. (d and e) Concentration-dependent effect of PD0325901 (IC_50_ = 17 nM) and GSK1120212 (IC_50_ = 4 nM). c-e: mean ± SD (n = 3).

Tregs (CD4^+^CD25^+^CD45RA^+^) were sorted from pre-enriched CD4^+^ cells from three healthy blood donors (Fig 1a) and stimulated with anti-CD3/CD28/CD2-coated MACSiBeads. Intracellular levels of FoxP3 expression were determined. The up-regulation of FoxP3 upon pan-T cell stimulation was blocked in the presence of MEKI (Fig 1b). Next, eight different MEKIs in various phases of clinical development were tested for their potency in preventing up-regulation of FoxP3 levels in sorted rTregs (Fig 1c). For two of the most potent MEKIs we examined their concentration-dependent effect and found that the non-ATP-competitive inhibitor PD0325901 and the ATP-competitive inhibitor GSK1120212 inhibited FoxP3 up-regulation during activation of rTregs with IC_50_ values of 17 and 4 nM, respectively (Fig 1d and 1e). Based on this, GSK1120212 was chosen for experiments with patient samples.

### Effect of MEKI on Treg activation in TB patient samples

**Fig 2:**
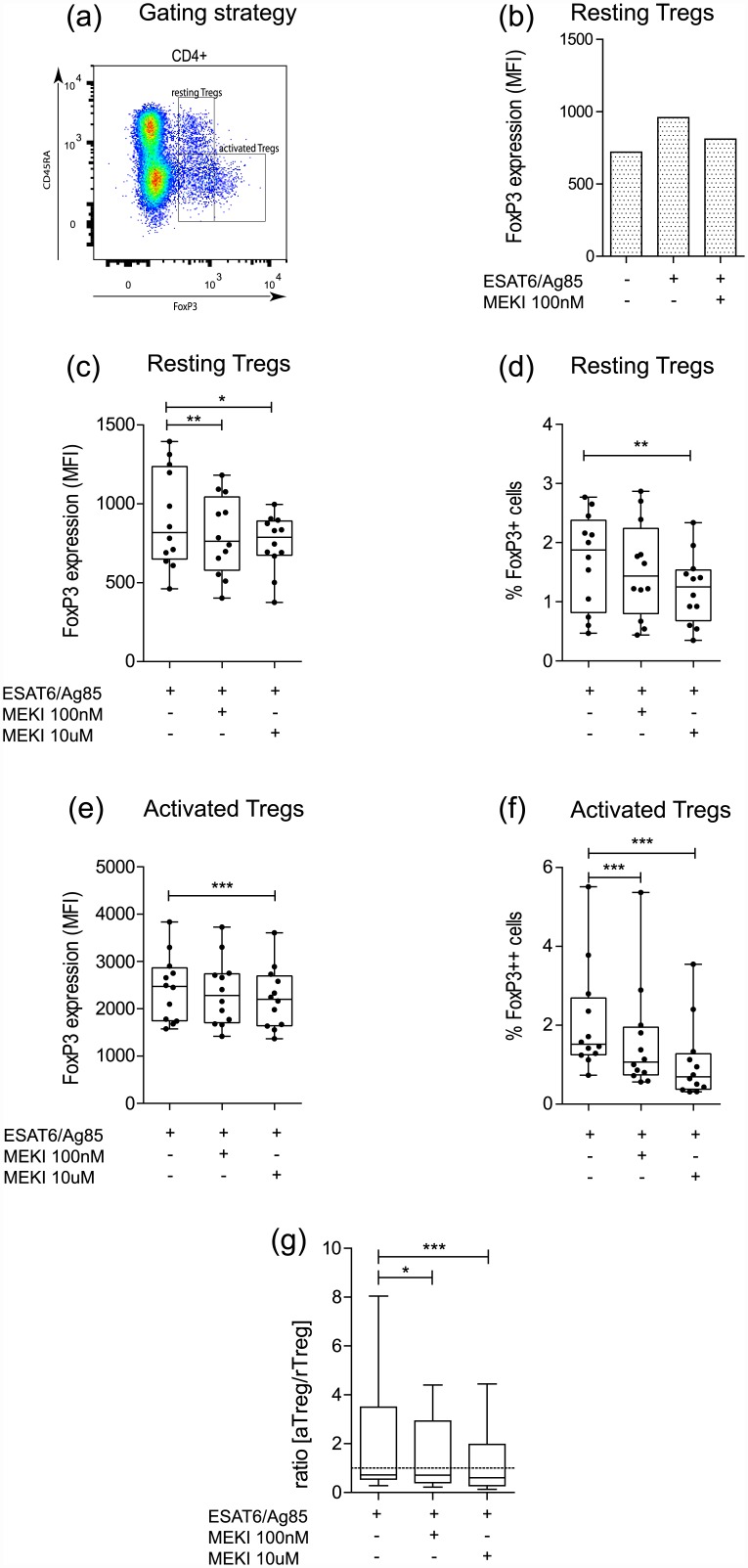
Effect of TB antigen and MEK inhibition on FoxP3 expression in resting and activated Tregs in TB patient samples. (a) Gating strategy for resting (CD4^+^CD45RA^+^FoxP3^+^) and activated (CD4^+^CD45RA^−^FoxP3^++^) Tregs. (b) PBMCs were stimulated with ESAT-6/Ag85 for 36 h in presence or absence of 100 nM MEK inhibitor GSK1120212 or left untreated. Effect of GSK1120212 (100 nM and 10 μM) on FoxP3 expression levels (MFI) (c, e) and number of FoxP3^+^ cells (%) (d, f) in resting (c, d) and activated (e, f) CD4^+^Tregs stimulated with ESAT-6/Ag85. (g) Ratio of aTreg over rTreg (%) in samples stimulated with ESAT-6/Ag85 alone and at two different MEKI concentrations as in d and e. (Boxes: median ± 25^th^ to 75^th^ percentile; whiskers: min to max, n = 12, * p< 0.05, ** p<0.01, *** p< 0.001).

We observed up-regulation of FoxP3 levels in rTregs in response to *Mtb*-specific antigens, which could be limited in the presence of MEKI (Fig 2a and 2b). MEK inhibition significantly decreased intracellular levels of FoxP3 both in rTregs (p <0.01) (Fig 2c) and aTregs (p <0.001) (Fig 2e) as well as the percentage of FoxP3^+^ rTregs (p <0.01) (Fig 2d) and FoxP3^++^ aTregs (p <0.001) (Fig 2f) and reduced the aTreg/rTreg ratio (Fig 2g).

### MEKI effect on TB specific CD4^+^ T cell cytokine responses

**Fig 3:**
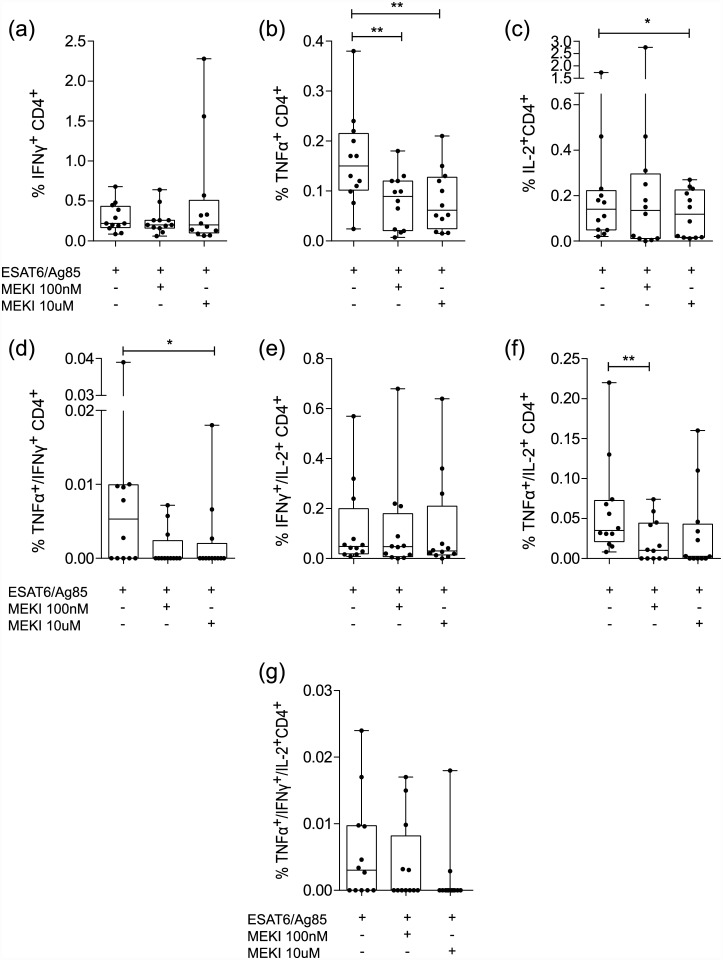
Effect of MEK inhibition on CD4^+^ T cell cytokine expression in response to TB antigens. PBMCs were stimulated with ESAT-6/Ag85 for 36 h in the presence or absence of MEK inhibitor GSK1120212 (100 nM, 10 μM) and the percentages of (a) IFN-γ^+^, (b) TNF-α^+^, (c) IL-2^+^, (d) TNF-α^+^/IFN-γ^+^, (e) IFN-γ^+^/IL-2^+^, (f) TNF-α^+^/IL-2^+^ and (g) TNF-α^+^/IFN-γ^+^/IL-2^+^ cells were determined by intracellular staining and FACS analysis. (Boxes: median ± 25^th^ to 75^th^ percentile; whiskers: min to max, n = 12, * p< 0.05,** p<0.01).

We next analyzed IFN-γ, TNF-α and IL-2 single, double and triple positive cells within the CD4^+^ T cell population of 12 patients with active TB (Fig 3). Both MEK concentrations significantly decreased the fraction of TNF-α^+^ cells (p <0.01) (Fig 3b), whilst IL-2^+^ (Fig 3c) and TNF-α^+^/IFN-γ^+^ cells (Fig 3d) were significantly reduced only at the highest MEK concentration (p <0.05) and TNF-α^+^/IL-2^+^ cells (Fig 3f) only at the lowest MEK concentration (p <0.01). No significant effect of MEK inhibition was observed on the fraction of IFN-γ^+^ cells (Fig 3a), IFN-γ^+^/IL-2^+^ cells (Fig 3e) or TNF-α^+^/IFN-γ^+^/IL-2^+^ cells (Fig 3g).

### Effect of MEKIs on Treg activation in HIV patient samples

**Fig 4:**
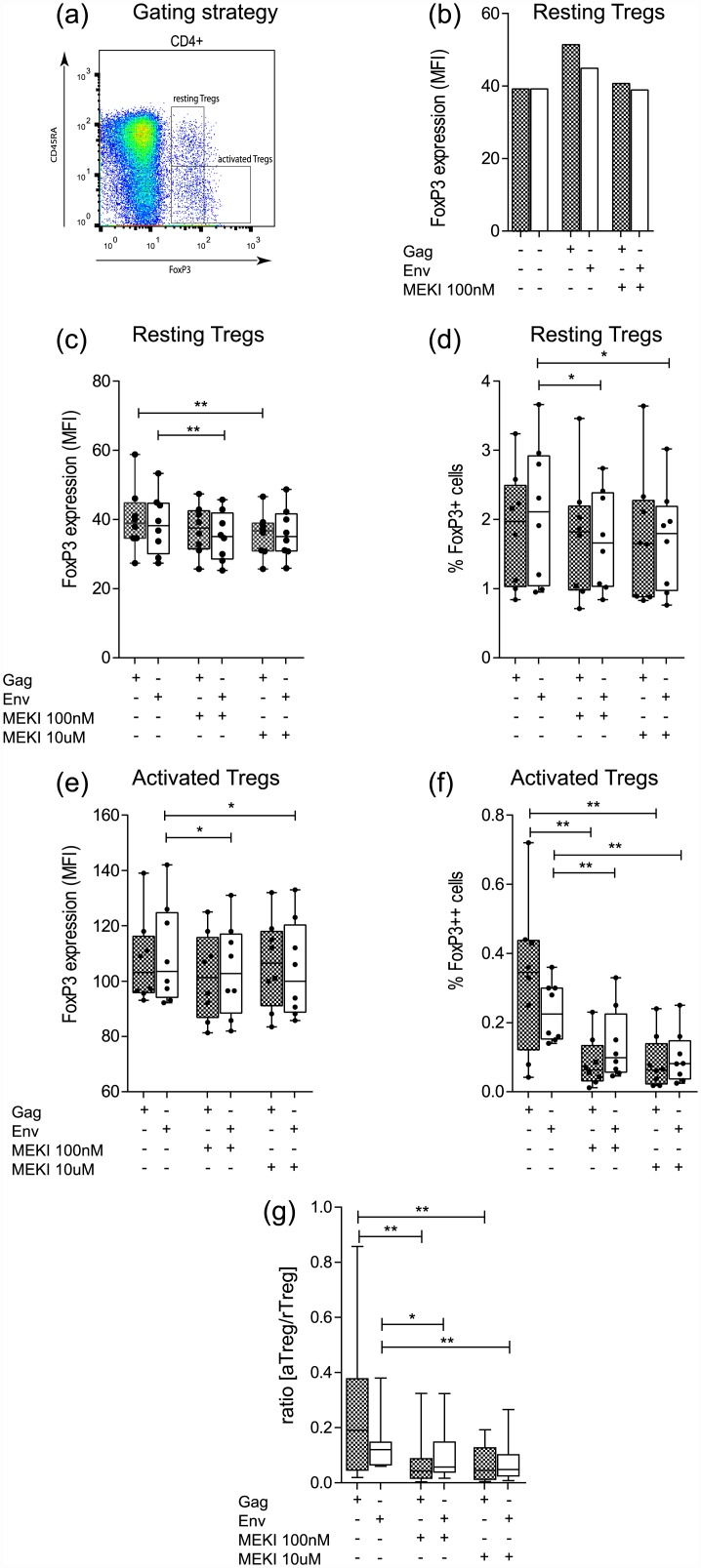
Effect of HIV antigen and MEK inhibition on FoxP3 expression in resting and activated Tregs in HIV patient samples. (a) Gating strategy for resting (CD4^+^CD45RA^+^FoxP3^+^) and activated (CD4^+^CD45RA-FoxP3^++^) Tregs. (b) PBMCs were stimulated with *Gag* (grey boxes) or *Env* (white boxes) for 36 h in presence or absence of MEK inhibitor GSK1120212 or left untreated. Effect of GSK1120212 (100 nM and 10 μM) on FoxP3 expression levels (MFI) (c, e) and number of FoxP3^+^ T cells (d, f) in rTregs (c, d) and aTregs (e, f) stimulated with *Gag* or *Env*. (g) Ratio of aTreg over rTreg (%) in samples stimulated with *Gag* or *Env* alone and at two different MEKI concentrations as in d and e. (Boxes: median ± 25^th^ to 75^th^ percentile; whiskers: min to max, n = 8,* p< 0.05, **p <0.01).

**Fig 5:**
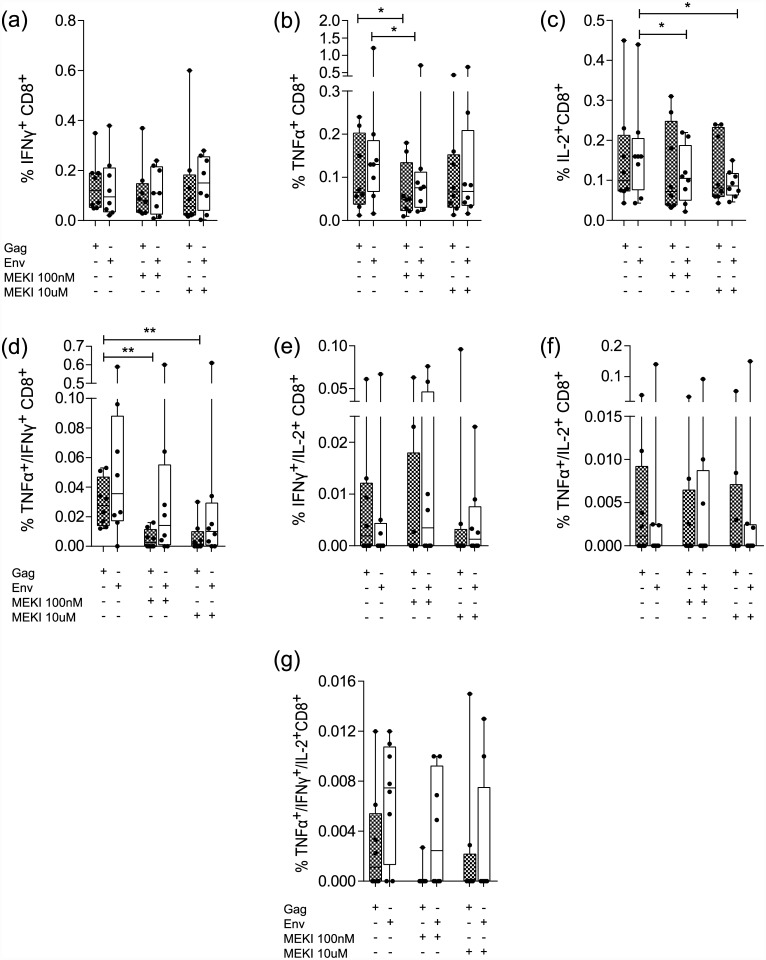
Effect of MEK inhibition on CD8^+^ T cell cytokine expression in response to HIV antigen *Gag* and Env. Patient PBMCs were stimulated with *Gag* (grey boxes) or *Env* (white boxes) for 36 h and the percentages of (a) IFN-γ^+^, (b) TNF-α^+^, (c) IL-2^+^, (d) TNF-α^+^/IFN-γ^+^, (e) IFN-γ^+^/IL-2^+^, (f) TNF-α^+^/IL-2^+^ and (g) TNF-α^+^/IFN-γ^+^/IL-2^+^ cells were determined by intracellular staining and FACS analysis.(Boxes: median ± 25^th^ to 75^th^ percentile; whiskers: min to max, n = 8, * p< 0.05,** p<0.01).

Since T cell activation varies substantially between HIV-infected patients and even within a given patient depending on stimulation with matrix (*Gag*) or envelope (*Env*) antigens, the effect of MEKIs on Treg activation was assessed separately in *Gag* or *Env* stimulated HIV samples (Figs 4 and 5). Moreover, two different concentrations of the MEKI GSK1120212 were tested. FoxP3 levels in rTregs decreased after stimulation in presence of MEKI at 10μM (p < 0.01) in *Gag* stimulated cells and at 100nM (p <0.01) in *Env* stimulated cells (Fig 4c). The percentage of rTregs decreased significantly after *Env* stimulation at both MEKI concentrations (p < 0.05), but not after *Gag* stimulation (Fig 4d). Similarly, FoxP3 levels in aTregs decreased significantly after *Env* stimulation at both MEKI concentrations (p <0.05), but not after *Gag* stimulation (Fig 4e). In contrast, the percentage of aTregs decreased significantly in both *Gag*- and *Env* stimulated samples at both MEKI concentrations (Fig 4f) as did the aTreg/rTreg ratios (Fig 4g).

### MEKI effect on HIV specific CD8^+^ T cell cytokine responses

HIV-specific IFN-γ, TNF-α and IL-2 cytokine responses and the effect of the MEKI were analyzed in the CD8^+^ T cell populations (Fig 5) TNF-α^+^ producing CD8^+^ T cells were reduced at low MEKI concentrations (p <0.05) in both *Gag* and *Env* stimulated cells (Fig 5b), whereas IL-2-producing cells were reduced at both inhibitor concentrations, but only in *Env* stimulated cells (p <0.05) (Fig 5c). In contrast, TNF-α^+^/IFN-γ^+^ cells were considerably reduced (p <0.01) at both MEKI concentrations only in *Gag-*stimulated cells (Fig 5d). MEK inhibition had no significant effect on the percentage of IFN-γ^+^, IFN-γ^+^/IL-2^+^, TNF-α^+^/IL-2^+^ or TNF-α^+^/IFN-γ^+^/IL-2^+^ CD8^+^ cells in either stimuli conditions (Fig 5a, 5e, 5f and 5g).

### MEKI effect on HIV specific CD4^+^ T cell cytokine responses

**Fig 6:**
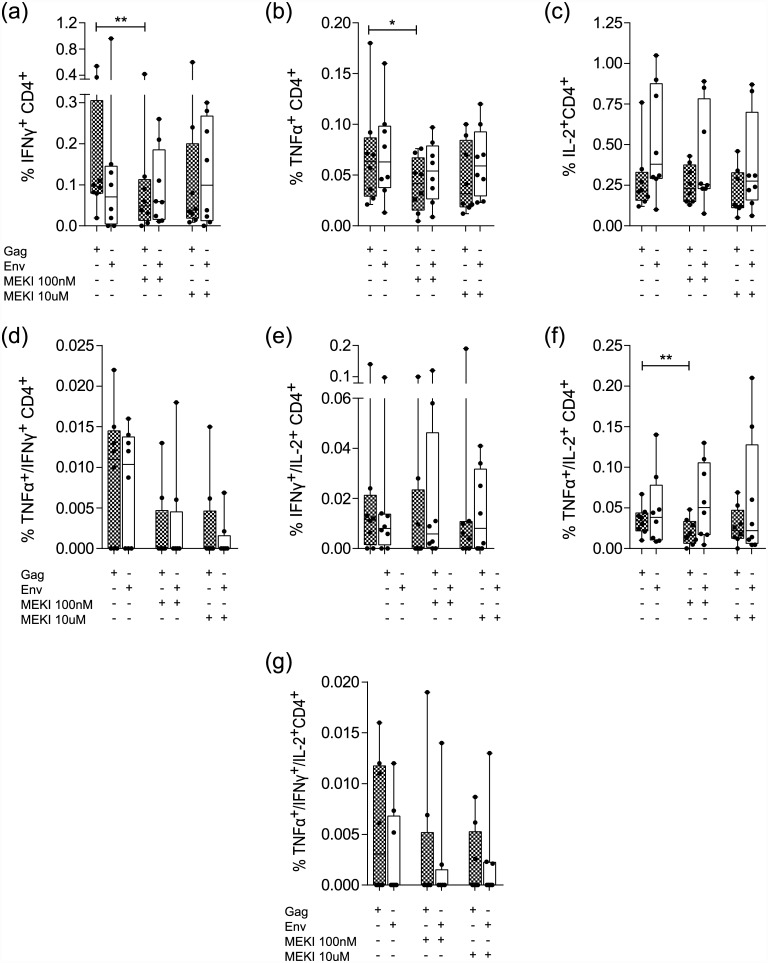
Effect of MEK inhibition on CD4^+^ T cell cytokine expression in response to HIV antigen *Gag* and *Env*. Patient PBMCs were stimulated with *Gag* (grey boxes) or *Env* (white boxes) for 36 h and the percentages of (a) IFN-γ^+^, (b) TNF-α^+^, (c) IL-2^+^, (d) TNF-α^+^/IFN-γ^+^, (e) IFN-γ^+^/IL-2^+^, (f) TNF-α^+^/IL-2^+^ and (g) TNF-α^+^/IFN-γ^+^/IL-2^+^ cells were determined by intracellular staining and FACS analysis. (Boxes: median ± 25^th^ to 75^th^ percentile; whiskers: min to max, n = 8, * p< 0.05, ** p<0.01).

HIV-specific IFN-γ, TNF-α and IL-2 cytokine responses and the effect of the MEKI were analyzed in the CD4^+^ T cell population (Fig 6). After *Gag*, but not *Env* stimulation a significant reduction of IFN-γ^+^ (p <0.01), TNF-α^+^ (p <0.05) and TNF-α^+^/IL-2^+^ (p < 0.01) producing cells was observed at low MEKI concentrations (Fig 6a, 6b and 6f). Other cytokine producing CD4^+^ T cell subsets showed no significant reduction (Fig 6b, 6d, 6e and 6g).

## Discussion

Different immune modulating therapies have been investigated in both HIV and TB infected patients with the aim of developing a more efficient host immune response [27, 28]. To our knowledge, we are the first to explore the effect of MEKIs on Tregs in these chronic infectious diseases. We show that targeting Tregs with MEKI significantly reduce levels and numbers of FOXP3^+^ Tregs in blood samples from both HIV and TB patients.

The FoxP3 transcription factor controls function and suppressive activity of Tregs [6, 29]. We show that low levels of FoxP3 in sorted rTregs of healthy blood donors increased upon activation. Adding MEKI prior to TCR stimulation resulted in an inhibitory effect on FoxP3 expression confirming that FoxP3 up-regulation depends on MEK/ERK signaling. This is in line with previous data where it was shown that MEK inhibited Tregs had reduced suppressive potential [10]. Other studies have shown that MEKIs have differential impact on T cell subsets, depending on their effector/memory stage [30]. This supports the notion that different T cell subpopulations depend on distinct signaling pathways for their activation [10, 31, 32] and might allow a selective interference with certain T cell subsets.

Tregs regulate and modify the immune responses in many infectious diseases, including TB and HIV [33]. In our study, MEK inhibition resulted in a clearly reduced up-regulation of FOXP3 in rTregs and aTregs in both TB and HIV peptide stimulated samples as well as reduced aTreg/rTreg ratios. The observed effect of MEKI on FoxP3 in rTreg can be explained by previous studies where we have shown by phospho-flow cytometry that although ERK phosphorylation is sustained in aTregs compared to rTregs or naive T cells, rTregs also have somewhat higher levels of ERK activation than naive T cells following cross-ligation (10). We also concede the possibility of additional indirect effects of MEKI on FoxP3. Hence, MEK activation in rTreg and ensuing regulation of FoxP3 can be modulated by MEKI.

In TB, studies have shown higher levels of circulating Tregs in patients with active TB than in subjects with latent TB infection [15, 34, 35]. Furthermore, it has been shown that Tregs delay the arrival of effector T cells in the lung during early infection [36] and prevent eradication of tubercle bacilli by suppressing otherwise efficient CD4^+^ T cell responses [37]. Consequently, the observed MEK induced reduction of Tregs could be beneficial in order to restore an effective immune response against *Mtb*.

The HIV positive patients in our study were all treatment naïve with stable CD4^+^ counts. Even though absolute numbers of CD4^+^ T cells are decreased in HIV infection, the relative frequency of Tregs is increased [19, 38]. However, the role of Tregs in HIV infection is still unclear. They can either have beneficial effects by suppressing generalized T cell activation or deleterious effects by weakening HIV-specific responses [39]. In immunological non-responders, it has been shown that increased Tregs are associated with sub-optimal CD4^+^ T cell recovery [40]. In our study, we demonstrate that HIV-peptide stimulated Tregs could be diminished by MEKIs, whereby the effects were more pronounced in the activated Treg subset as evident from the fact that the ratio of aTreg over rTreg was reduced. Furthermore, an *in vitro* study showed a differential effect of MEK/ERK pathway inhibition on viral replication which was dependent on the HIV co-receptor tropism (X4 or R5) [41]. This indicates specificity of MEKIs in their contribution to viral control.

Our study was set up to evaluate the effect of MEKI in TB and HIV patient samples but not to analyze differences between the groups. It was however notable that the ratio between resting and activated Tregs differed between HIV and TB patients. HIV patients exhibit reduced numbers of aTregs compared to rTregs in our study. This agrees with other studies where significantly reduced numbers of aTregs have been observed during HIV infection [42, 43]. In active pulmonary TB the majority of circulating Tregs were shown to exhibit an activated CD45RO^+^ phenotype [44], however to our knowledge there are no other studies on untreated TB patients that report aTreg and rTreg on the basis of CD45RA expression. As we used CD25 and CD45RA to identify activated and naïve cells we also examined whether levels of either marker were affected by treatment with MEKI in the TB (S1 Fig) and HIV (S2 Fig) patient samples and found that they were not.

The effects of stimulation with different disease-specific peptide antigens can differ between patients, depending on disease stage, type of pathogen strain as well as the immune status of the patient at the time of blood sampling. This might explain differences in the effect range of TB and HIV-specific stimulations at different MEK inhibitor concentrations. There were also differences in the MFI FoxP3 subsets between the HIV and TB samples, but the interpretation of differences in MFI values are limited due to methodological factors (HIV and TB samples were analyzed at different time points with two different flow cytometers).

Cytokine producing CD4^+^ and CD8^+^ T cells play an important role in the protective immune responses against both *Mtb* and HIV [45, 46], but still there are controversies concerning the definition of correlates of protection [47–49]. By analyzing the effects of MEKIs on cytokine producing CD4^+^ and CD8^+^ T cells we found a significant reduction in TNF-α^+^ and IFN-γ^+^/TNF-α^+^ in both TB (CD4^+^ cells) and HIV (CD8^+^ and CD4^+^) samples. In addition MEK inhibition significantly reduced IL-2^+^/TNF-α^+^ and IL-2^+^ CD4^+^ T cells in TB patients.

The observed reduction of cytokines in our study contradicts the hypothesis of a more enhanced pro-inflammatory T cell response following a reduction in numbers of Tregs in TB and HIV patients. However, a decrease in IFN-γ^+^ and IL-17^+^ CD4^+^ T cells as well as IFN-γ^+^ and TNF-α^+^ CD8^+^ T cells after PMA-ionomycin stimulation in the presence of MEKI was shown in studies with healthy individuals [50]. Furthermore, it is known that untreated TB and HIV patients feature a hyper-activated immune system with a dysregulated network of pro- and anti-inflammatory cytokines [51–53], which also applies to the subjects in our study and may have contributed to less efficient Th1 effector responses following reduced numbers of Tregs. There may also be an inhibitory effect of MEKIs not only in the MEK/ERK signaling pathway in Tregs, but also in the intracellular pathway of different effector T cell subsets [30]. In both the HIV and TB samples the effect of MEK inhibition was most prominent in TNF-α single and double positive cells, which could be explained by TNF-α being regulated by the NFAT1 transcription factor downstream of the MEK/ERK [54].

Pro- inflammatory cytokines, among them TNF-α, damage lymphoid tissue in HIV infection, resulting in a decline of regenerative capacity and loss of effective anti-HIV immunity [11]. In HIV infection, high levels of TNF-α are present at all stages of chronic infection [55] and this has been associated with increased viral replication and destruction of infected CD4^+^ T cells [56]. It has been shown that during efficient ART, levels of IL-2 mRNA increase and IFN-γ mRNA as well as TNF-α levels decrease [57]. Further, persistence of increased TNF-α levels after initiation of ART is correlated with virological and immunological treatment failure [58]. Treatment of HIV infected patients with TNF inhibitors resulted in lower viral load [59, 60] and inhibition of HIV-1 replication [61], however there was no effect on CD4^+^ T cell counts or patient survival. Also IFN-γ plays a role in inducing inflammation as well as antiviral immunity. IFN-γ is detected in the acute phase of infection and has an influence on the viral load set point [62]. Furthermore, IFN-γ in addition to other cytokines is produced in response to HIV-specific stimulation of CD4^+^ T cells [63]. IL-2 has been used as an adjuvant in HIV treatment; however, despite an increase in CD4^+^ counts no clinical benefit was observed [64].

In TB, studies exploring recombinant IL-2 as adjunctive therapy to standard TB treatment have shown contrasting results; one study reported improvement of clinical symptoms with recombinant IL-2 in addition to multidrug TB therapy [65], whilst another study revealed rather detrimental effects of adjunctive IL-2 therapy [66], the latter assumed to be due to IL-2 mediated expansion of Tregs [67]. Excessive levels of TNF-α also play a role in TB pathogenesis leading to a more favorable growth permissive milieu for the mycobacteria [68], even though the use of TNF-α inhibitors as an immune modulating treatment increases the risk of reactivation of latent TB [69, 70]. Thus, in the context of chronic TB disease it is likely that a reduction of TNF-α, but not a complete block, would facilitate the containment and eradication of *Mtb*. We have recently reported an early decrease of pro-inflammatory cytokines associated with an early temporary increase in Tregs during effective anti-TB treatment [71]. In conclusion, dampening of the pro-inflammatory cytokines as shown in our study may be beneficial to the host depending on the stage of infection and disease.

The stage of disease in individual patients is central to the issue of the benefit of immune modulating agents as adjunctive therapy in infectious diseases. Some patients may actually require an increase in inflammatory responses, whereas others require dampening of the immune response with the aim of refocusing the immune response toward clinically and biologically relevant immunoreactivity [72]. MEKIs may have therapeutic potential by blocking Treg activation despite their inhibitory effect on pro-inflammatory cytokines, but these effects on effector T cells could also be a problem from a therapeutic perspective. This was the first study in untreated HIV and TB patients and therefore further evaluation is needed.

We acknowledge that the small number of patients in our study increases the risk of not detecting effects of the MEKI that could present with increased sample size, but we have limited the discussion to the significant findings. Our study population consisted of patients with untreated TB and HIV, both groups generally known to be characterized by a state of hyper-activated immune responses.

In conclusion, we were able to modulate disease-specific Tregs in TB and HIV patient samples by decreasing the numbers of rTreg and aTreg as well as inhibiting activation of Tregs by preventing FoxP3 up-regulation with MEKI. MEK inhibition also reduced *in vitro* stimulated T cell cytokine production. However, we postulate that in a state of chronic immune activation a decrease in pro-inflammatory cytokines may actually represent a beneficial response to the host. This study provides important and novel data on the *in vitro* effect of MEKIs in two different chronic infectious diseases. Whether MEKIs can be used as a supplement to current HIV or TB therapies needs further investigation in patients in different stages of these chronic infectious diseases, during microbe specific treatment and in combination with vaccination strategies.

## Supporting Information

S1 Fig Effect of MEK inhibition on CD25 and CD45RA expression in CD4^+^ T cells in response to TB antigens. PBMCs were stimulated with ESAT-6/Ag85 for 36 h in the presence or absence of MEK inhibitor GSK1120212 (100 nM, 10 μM) and the percentages of (a) CD4^+^CD25^+^ and (b) CD4^+^CD45RA^+^ were determined. (Boxes: median ± 25^th^ to 75^th^ percentile; whiskers: min to max, n = 12).
(EPS)

S2 Fig Effect of MEK inhibition on CD25 and CD45RA expression in CD4^+^ T cells in response to HIV antigens. PBMCs were stimulated with Gag (grey boxes) or Env (white boxes) for 36 h in the presence or absence of MEK inhibitor GSK1120212 (100 nM, 10 μM) and the percentages of (a) CD4^+^CD25^+^ and (b) CD4^+^CD45RA^+^ were determined. (Boxes: median ± 25^th^ to 75^th^ percentile; whiskers: min to max, n = 8).
(EPS)

## Abbreviations

ART: antiretroviral therapy
aTregs: activated Tregs
FoxP3: Forkhead box P3
HIV: Human immunodeficiency virus
MEKI: MEK inhibitor (MEK/ERK signaling pathway inhibitor)
MFI: median fluorescent intensity
Mtb: Mycobacterium tuberculosis
rTregs: resting Tregs
TB: Tuberculosis
Tregs: Regulatory T cells
